# Variational Quantum Chemistry Programs in JaqalPaq

**DOI:** 10.3390/e23060657

**Published:** 2021-05-24

**Authors:** Oliver G. Maupin, Andrew D. Baczewski, Peter J. Love, Andrew J. Landahl

**Affiliations:** 1 Department of Physics and Astronomy, Tufts University, Medford, MA 02155, USA; oliver.maupin@tufts.edu (O.G.M.); peter.love@tufts.edu (P.J.L.); 2 Sandia National Laboratories, Center for Computing Research, Albuquerque, NM 87185, USA; adbacze@sandia.gov; 3 Center for Quantum Information and Control, University of New Mexico, Albuquerque, NM 87131, USA; 4 Department of Physics and Astronomy, University of New Mexico, Albuquerque, NM 87131, USA; 5 Computational Science Initiative, Brookhaven National Laboratory, Upton, NY 11973, USA

**Keywords:** quantum computing, quantum simulation, NISQ algorithms, quantum chemistry, ion trap quantum computing, quantum software

## Abstract

We present example quantum chemistry programs written with JaqalPaq, a python meta-programming language used to code in Jaqal (Just Another Quantum Assembly Language). These JaqalPaq algorithms are intended to be run on the Quantum Scientific Computing Open User Testbed (QSCOUT) platform at Sandia National Laboratories. Our exemplars use the variational quantum eigensolver (VQE) quantum algorithm to compute the ground state energies of the $H_{2}$, ${\mathrm{HeH}}^{+}$, and LiH molecules. Since the exemplars focus on how to program in JaqalPaq, the calculations of the second-quantized Hamiltonians are performed with the PySCF python package, and the mappings of the fermions to qubits are obtained from the OpenFermion python package. Using the emulator functionality of JaqalPaq, we emulate how these exemplars would be executed on an error-free QSCOUT platform and compare the emulated computation of the bond-dissociation curves for these molecules with their exact forms within the relevant basis.

## 1. Introduction

Welcome quantum computer programmer! Jaqal, an acryonym for Just Another Quantum Assembly Language, is a quantum assembly language that was “designed by quantum information scientists, for quantum information scientists.” Due to this, Jaqal enables scientists to explore near-term quantum testbeds using language constructs that make explicit connections to hardware primitives. In particular, Jaqal allows programmers to schedule parallel and sequential gate blocks, associate gates to underlying pulse programs, and bind qubit variables to hardware qubit carriers [1]. Jaqal was developed within the QSCOUT (Quantum Scientific Computing Open User Testbed) project at Sandia National Laboratories. For a detailed specification of the Jaqal language, please see [2].

While programmers can code directly in Jaqal if they wish to do so, it can be more convenient to use the associated python JaqalPaq meta-programming package instead. JaqalPaq allows programmers to combine classical python instructions with quantum Jaqal instructions to generate rich and expressive programs. JaqalPaq provides functionalities to do the following:

- Parse Jaqal text files into Jaqal quantum circuit objects.
- Manipulate Jaqal quantum circuit objects using python.
- Emulate the behavior of Jaqal quantum circuit objects.
- Output Jaqal text files from Jaqal quantum circuit objects.

Programmers who have experience with other quantum assembly languages may want to use the JaqalPaq-extras python package. The JaqalPaq-extras package provides methods for parsing and transpiling quantum assembly code written in other languages into Jaqal circuit objects relevant for the QSCOUT platform. Currently, the JaqalPaq-extras package supports transpiling from the following languages: QISkit (IBM), Cirq (Google), Quil (Rigetti), t|ket〉 (Cambridge Quantum Computing), and ProjectQ (ETH Zurich).

After briefly covering how to install the relevant python packages in Section 2, we review the variational quantum eigensolver (VQE) [3] algorithm in Section 3. We then present and explain three exemplars written using JaqalPaq in Section 4, Section 5 and Section 6. These three programs each estimate the ground-state energy of a small molecule using the VQE algorithm [3,4,5]. The molecules explored by these exemplars are small enough that they can be executed on the first-generation QSCOUT quantum computer, which can run quantum circuits on up to three qubits. After this, in Section 7, we describe a variant of one of the exemplars that could be executed on an envisioned future QSCOUT platform that can operate on at least four qubits.

The intended audience for this document is beginner JaqalPaq programmers who may or may not be experts in quantum chemistry but who have some familiarity with quantum circuits and how variational quantum algorithms work, at the level covered in [4,5,6,7]. To that end, we do not delve deeply into the underlying quantum chemistry minutiae needed to give meaning to these exemplars. For example, we offload the evaluation of the integrals needed to compute the Hamiltonian coefficients for the molecules and the subsequent fermion-to-qubit mapping to the PySCF and OpenFermion python packages, respectively.

We also do not analyze the merits and flaws of the various basis sets used to second-quantize molecular Hamiltonians. Instead, we simply assert which basis we are using. Finally, we do not detail the fermion-to-qubit mappings and effective Hamiltonian reductions that we use, instead referring the reader to the relevant literature where these topics are discussed. It is our hope that by streamlining the discussion in this way that programmers will be able to distill the essence of how JaqalPaq can be used to run variational quantum algorithms, enabling them to write their own algorithms and explore the questions they wish to investigate.

As a brief summary, Table 1 lists each of the molecules covered by exemplars in this document, what the relevant atomic parameters are for the corresponding VQE algorithm, and which section in the document it is discussed in (with a hyperlink).

We note that all of the exemplars described in this paper are available under the examples directory of the JaqalPaq git repository at https://gitlab.com/jaqal/jaqalpaq (accessed on 23 May 2021). These simulations are not prohibitively intensive, taking on the order of an hour to run on a modern laptop.

## 2. Setup and Installation of JaqalPaq

To begin working with our exemplars, one first needs to install OpenFermion, PySCF, OpenFermionPySCF, QSCOUT-gatemodels, pyGSTi [8,9], and JaqalPaq. We briefly explain how to do this in this section.

### 2.1. Generic Notes on the Installation Procedure

Throughout, we provide installation instructions using the pip package-management system in both the development and user modes. Development mode installation requires the retrieval of the package source code itself and the subsequent installation of the package relative to a particular working directory for which the user has adequate permissions. In the development mode instructions below, git is used to clone the source on the user’s machine through HTTPS, and the working directory relative to which the package is installed is the cloned source.

This option is preferred for users who intend to make modifications to the source code of any of the packages being installed. In contrast, user mode installation does not require the user to first retrieve the package source, and the installation of the package will be relative to the user’s home directory. This option is preferred for users who do not intend to modify the source code of any of the packages being installed. Naturally, it is possible to install some packages in development mode and some packages in user mode.

### 2.2. Installing OpenFermion

OpenFermion is a python library used to manipulate quantum chemistry Hamiltonians; we use it to reduce the fermionic Hamiltonians of our molecules and transform them to act on qubits. Documentation is available at https://github.com/quantumlib/OpenFermion (accessed on 23 May 2021). To install the latest version of OpenFermion in development mode:

cd <install directory>

git clone https://github.com/quantumlib/OpenFermion

cd OpenFermion

python -m pip install -e .

To install the latest Python Package Index (PyPI) release as a library in user mode:

python -m pip install --user openfermion

### 2.3. Installing PySCF

PySCF is a second python library that we use to calculate the coefficients of the fermionic Hamiltonians of our molecules at varying bond lengths. Documentation is available at http://pyscf.org/quickstart.html (accessed on 23 May 2021). The simplest way to install PySCF is via the Python Package Index (PyPI), which provides a precompiled PySCF code (python wheel) that works on most Linux systems, macOS systems, and Ubuntu subsystems on Windows 10:

python -m pip install pyscf

If you already have pyscf installed, you can upgrade it to the newest version:

python -m pip install --upgrade pyscf

### 2.4. Installing OpenFermionPySCF

OpenFermionPySCF is a third python library that allows OpenFermion and PySCF to interface directly; we use it to invoke and execute PySCF calculations, the results of which are turned into data structures accessible by OpenFermion. We once again refer to the documentation available at https://github.com/quantumlib/OpenFermion-PySCF (accessed on 23 May 2021). To install the latest versions of OpenFermion and OpenFermion-PySCF in development mode:

cd <install directory>

git clone https://github.com/quantumlib/OpenFermion-PySCF

cd OpenFermion-PySCF

python -m pip install -e .

Alternatively, to install the latest PyPI releases as libraries in user mode:

python -m pip install --user openfermionpyscf

### 2.5. Installing JaqalPaq

JaqalPaq can also be installed using pip, and documentation is available at https://gitlab.com/jaqal/jaqalpaq (accessed on 23 May 2021). To install JaqalPaq in development mode:

cd <install directory>

git clone https://gitlab.com/jaqal/jaqalpaq

cd jaqalpaq

python -m pip install -e .

Alternatively, to install it in user mode:

python -m pip install --user jaqalpaq

We also note the availability of transpilers from other common quantum assembly languages to Jaqal circuit objects in JaqalPaq. These can be installed in development mode using:

cd <install directory>

git clone https://gitlab.com/jaqal/jaqalpaq-extras

cd jaqalpaq-extras

python -m pip install -e .

Alternatively, in user mode:

python -m pip install --user jaqalpaq-extras

### 2.6. Installing pyGSTi

JaqalPaq has two further dependencies that also require installation. QSCOUT-gatemodels and pyGSTi, the latter being a dependency of the former, and thus we discuss its installation first. Documentation for pyGSTi is available at https://github.com/pyGSTio/pyGSTi (accessed on 23 May 2021). It can be installed in development mode using:

cd <install directory>

git clone https://github.com/pyGSTio/pyGSTi.git

cd pyGSTi

python -m pip install -e .

Alternatively, in user mode:

python -m pip install --user pygsti

We note that pyGSTi has numerous optional dependencies that can also be installed using pip and refer the user to https://github.com/pyGSTio/pyGSTi (accessed on 23 May 2021) for more information.

### 2.7. Installing QSCOUT-Gatemodels

The other remaining JaqalPaq dependency is the gate pulse file that defines the gates that JaqalPaquses. Documentation can be found at https://gitlab.com/jaqal/qscout-gatemodels (accessed on 23 May 2021). It can be installed in development mode using:

cd <install directory>

git clone https://gitlab.com/jaqal/qscout-gatemodels

cd qscout-gatemodels

python -m pip install -e .

Alternatively, in user mode:

python -m pip install --user qscout-gatemodels

## 3. Brief Review of the Variational Quantum Eigensolver (VQE) Algorithm

Quantum chemistry was identified as a promising application of future quantum computers in  [10]. The variational quantum eigensolver (VQE) algorithm was later developed as an approach to quantum chemistry problems suitable for NISQ (Noisy Intermediate-Scale Quantum) computers. A key advantage of the VQE algorithm is that it can be run on near-term quantum hardware without the need for large-scale fault-tolerant quantum computing architectures facilitated by quantum error-correcting codes [3,4].

We note that, in recent results [11], VQE may not show a quantum advantage over classical computational chemistry methods; however, its use here as an exemplary algorithm is still of interest. The VQE algorithm is a hybrid quantum-classical algorithm that uses a classical optimizer to minimize a cost function, which is evaluated using measurement outcomes from circuits executed on quantum hardware [3,4]. In the context of the chemistry exemplars in this document, this cost function is the ground-state energy of the electronic Hamiltonian of a small molecule. The VQE algorithm relies on an ansatz, which is a space of quantum states that are parametrized by a vector of classical variables $\vec{\theta }=\left(\theta_{1},\theta_{2},\ldots ,\theta_{n}\right)$. The VQE algorithm uses this ansatz to construct and execute a quantum circuit that prepares a trial state within that space.

**Figure 1:**
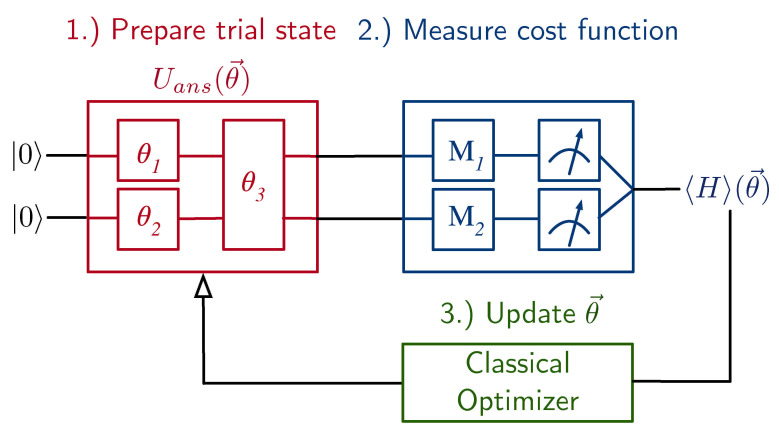
An exemplar VQE circuit with two qubits and an ansatz, $U_{ans}\left(\vec{\theta }\right)$, parameterized by the angles $\vec{\theta }=\left(\theta_{1},\theta_{2},\theta_{3}\right)$. (**1.**) The circuit prepares an ansatz state parameterized by angles $\vec{\theta }$. (**2.**) Different circuits measure the expectation values of terms in the Hamiltonian to calculate the cost function. (**3.**) The value of the cost function and angles $\vec{\theta }$ are fed into a classical optimizer to compute new angles $\vec{\theta^{\prime }}$.

For a given trial state prepared on quantum hardware, the expectation value of the cost function, $\langle H\rangle \left(\vec{\theta }\right)$, is estimated from the outcomes of a set of Pauli measurements. The estimate and the vector of parameters are then fed into a classical optimizer that produces a new vector of parameters $\vec{\theta^{\prime }}=\left(\theta_{1}^{\prime },\theta_{2}^{\prime },\ldots \theta_{n}^{\prime }\right)$ in order to compute a new value of the cost function, and so on. The optimizer iterates this process, with each step guiding the value of the vector of parameters closer to the ansatz state that minimizes the cost function. For our chemistry exemplars, this corresponds to the electronic ground state energy for a fixed molecular geometry. A simplistic version of this process is shown in Figure 1.

The VQE algorithm can be separated into three primary components. The first is a quantum circuit that prepares an ansatz state. There is a correspondence between states in the span of the ansatz and a circuit that generates each of them. This is illustrated in Figure 1 for a two-qubit example in terms of a unitary operation, $U_{ans}\left(\vec{\theta }\right)$, which transforms $|00\rangle$ into an ansatz state. The unitary operation $U_{ans}\left(\vec{\theta }\right)$ is itself compiled into two single-qubit gates and one two-qubit gate, each of which is parametrized by one of the classical variables in $\vec{\theta }$. By adjusting the value of $\vec{\theta }$, one can produce different states within the ansatz with the intent of finding the one for which the cost function is minimized. For the examples in this document, we used the Unitary Coupled Cluster ansatz with single and double excitations (UCCSD) [12,13,14,15].

The second piece of the VQE algorithm is one or more circuits that perform Pauli measurements of the prepared trial state to estimate the expectation value of the cost function. This cost function will be comprised of products of Pauli operators. For each of these terms, a separate circuit will prepare the ansatz state and rotate it to the correct basis and perform a measurement. Repeated sampling of these circuits will then allow us to calculate the expectation value of the cost function as a whole. Depending on the function one seeks to minimize, a great deal of optimization may go into this component of the VQE algorithm, and there are many techniques available for reducing the number of terms that must be measured [16,17,18,19,20].

The third piece of the VQE algorithm is the classical optimizer. A wide variety of classical optimizers may be used, although their efficiency varies; the best practices remain an active area of research [21,22,23]. The optimizer takes, as inputs, the expectation values of each of the terms in the cost function and their respective weights, as well as the vectors of the parameters that produced it, and outputs a new vector of parameters that ideally produces a state that is closer to minimizing the cost function. For each iteration in this loop, a new ansatz circuit must be created to account for the changing vector of parameters; however, the measurement circuits typically do not change.

## 4. $H_{2}$: Molecular Hydrogen

In this Section, we describe our first example VQE algorithm: computing the ground-state energy of molecular hydrogen within the UCCSD ansatz. Like the *Drosophila* fruit fly is to biology research, this is the “Hello world” program of the field, as it was the first example of quantum computation applied to quantum chemistry [24]. It has also been the subject of many experimental realizations [25,26,27,28]. By being so simple, this example lacks many features that larger problems possess, such as contextuality [29,30]. Nonetheless, it is the simplest possible quantum calculation of the ground-state energy of a molecule, and is, therefore, a natural starting point.

### 4.1. $H_{2}$: Derivation of the Hamiltonian

Our process for computing the ground state energy of $H_{2}$ closely follows the works of O’Malley et al. [25] and Hempel et al. [28]. As shown in those works, the second-quantized fermionic Hamiltonian for $H_{2}$ represented in the STO-3G basis set can be encoded to act on four qubits (indexed 0–3) using the Bravyi–Kitaev mapping [31]. Doing so will result in an expression that acts with the Pauli operators *I* and *Z* on the qubits 1 and 3 and the Pauli operators *X* and *Y* on the qubits 0 and 2. States within the UCCSD ansatz are generated from the Hartree–Fock state, which is a single computational basis state in the Bravyi–Kitaev encoding. The UCCSD ansatz is generated by single- and double-excitation operators that include strings of fermionic operators that also appear in the Hamiltonian.

The states of qubits 1 and 3 are left unchanged by the application of the ansatz generating circuit. Thus, we can reduce the dimensionality of the problem by projecting onto the subspace in which qubits 1 and 3 both take a fixed value—this is known as tapering [29]. It turns out that the ground state for the UCCSD ansatz is in the subspace in which both qubits are in the |0〉 state. Therefore, the VQE algorithm can be reformulated to find the ground state of an effective Hamiltonian that only acts on the qubits 0 and 2, which will, henceforth, be re-indexed as 0 and 1, as was done in  [25]. Specifically, the reduced Hamiltonian is [25]:

(1) $$H_{BK}=c_{0}I+c_{1}Z_{0}+c_{2}Z_{1}+c_{3}Z_{1}Z_{0}+c_{4}X_{1}X_{0}+c_{5}Y_{1}Y_{0}.$$

For this reduced Hamiltonian, the UCCSD ansatz becomes a space of two-qubit states parameterized by a single real variable. The Hartree–Fock reference state from which this space is generated is the two-qubit computational basis state for which the expectation value of Equation (1) is smallest. It is easy to determine which state this is by inspection. The identity acts on all basis states in the same way, and thus the value of $c_{0}$ is irrelevant. The expectation values of the $X_{1}X_{0}$ and $Y_{1}Y_{0}$ terms are zero for any basis state; therefore, the values of $c_{4}$ and $c_{5}$ are also irrelevant. The sign of $c_{1}$ is positive, and $c_{2}$ is negative; thus, these will reduce the energy expectation value when the qubits 0 and 1 are in the states 1 and 0, respectively. Finally, the sign of $c_{3}$ is positive, and thereby the energy expectation value will be reduced when the qubits 0 and 1 have odd parity. Thus, we can see that the Hartree–Fock state is $|\psi_{HF}\rangle =|01\rangle$, by inspection. To be clear about the ordering, this means that qubit 0 is in the |1〉 state, and qubit 1 is in the |0〉 state.

For each term in the reduced Hamiltonian in Equation (1), we must create a Jaqal circuit to measure the expectation value of that term acting on the VQE ansatz state. One way to write this with JaqalPaq is as follows: (The python import and other header statements are not listed below to maintain the focus on the relevant code. The full python code for this example can be found in Appendix A and in the JaqalPaq distribution under the jaqalpaq/examples directory.)

# Define Pauli strings that appear in the reduced two-qubit Hamiltonian

#          q0 ,  q1

terms = [[None,None],[’Z’,None],[None,’Z’],[’Z’,’Z’],[’X’,’X’],[’Y’,’Y’]]

# Calculate effective coefficients for the reduced two-qubit Hamiltonian

# from those of the four-qubit Hamiltonian.

# Derivation follows arXiv:1803.10238v2 appendix A-2

fs = hamiltonian_bk.terms #Old coefficients from OpenFermion Hamiltonian

c0 = (fs[()] + fs[(1, ’Z’),] + fs[(1, ’Z’), (3, ’Z’),]).real

c1 = (fs[(0, ’Z’),] + fs[(0, ’Z’), (1, ’Z’),]).real

c2 = (fs[(2, ’Z’),] + fs[(1, ’Z’), (2, ’Z’), (3, ’Z’),]).real

c3 = (fs[(0, ’Z’), (2, ’Z’),] + fs[(0, ’Z’), (1, ’Z’), (2, ’Z’),]

+ fs[(0, ’Z’), (2, ’Z’), (3, ’Z’),]

+ fs[(0, ’Z’), (1, ’Z’), (2, ’Z’), (3, ’Z’)]).real

c4 = (fs[(0,’X’), (1, ’Z’), (2, ’X’),]

+ fs[(0, ’X’), (1, ’Z’), (2, ’X’), (3, ’Z’),]).real

c5 = (fs[(0, ’Y’), (1, ’Z’), (2, ’Y’),]

+ fs[(0, ’Y’), (1, ’Z’), (2, ’Y’), (3, ’Z’),]).real

#New coefficients are linear combinations of old coefficients

cs = [c0, c1, c2, c3, c4, c5]

for i in range(len(terms)):

builder = circuitbuilder.CircuitBuilder(

native_gates=normalize_native_gates(native_gates.NATIVE_GATES))

# Create a qubit register

q = builder.register(’q’, 2)

# Define a hadamard macro

hadamard = circuitbuilder.SequentialBlockBuilder()

hadamard.gate(’Sy’, ’a’)

hadamard.gate(’Px’, ’a’)

builder.macro(’hadamard’, [’a’], hadamard)

#################

#Apply UCC Ansatz

#################

# Change basis for measurement depending on term

for j, qubit in enumerate(terms[i]):

if qubit == ’X’:

builder.gate(’hadamard’, (’array_item’, q, j)),

if qubit == ’Y’:

builder.gate(’Sxd’, (’array_item’, q, j)),

builder.gate(’measure_all’)

circuit = builder.build()

# Format results of simulation as a list of lists

#Run circuit on emulator

sim_result = emulator.run_jaqal_circuit(circuit)

#Extract probabilities

sim_probs = sim_result.subcircuits[0].probability_by_int

#Combine lists of probs of each term in Hamiltonian

term_probs += [sim_probs]

This code begins with a manual definition of the terms and coefficients of our reduced two-qubit Hamiltonian. It then creates six different Jaqal circuit expressions, one for each Pauli term in the Hamiltonian. Each expression begins by creating a two-qubit register and defining a macro for a Hadamard gate. Then, each circuit expression ends with a different measurement, depending on its corresponding Pauli term. This code will append gates to each circuit to map the Pauli term to the logical *Z* basis before measurement. For the *X* basis, we implement a Hadamard as a $\frac{\pi }{2}$ rotation about the *y*-axis followed by a π rotation about the *x*-axis. For the *Y* basis, we simply rotate about the *x*-axis by $\frac{\pi }{2}$. Lastly, the circuit expression is made into a circuit object by the circuitbuilder class.

This Jaqal circuit, beginning with the preparation of the qubits through to their measurement, is run on the emulator to determine the probabilities of certain output states and, from that, the expectation values of each of the terms in the Hamiltonian. The expectation values are then weighted by coefficients $c_{i}$ derived from the original Hamiltonian coefficients $f_{i}$ that were calculated using PySCF [32]. We note that using the emulator in this way to access the probabilities directly is impossible in the experiment; therefore, we also included code that shows how to estimate these probabilities by repeatedly sampling the output of a circuit, as would be done on the QSCOUT hardware:

# Format results of simulation as a list of lists

probs = np.zeros(4) # Number of possible states

#Run circuit on emulator

sim_result = emulator.run_jaqal_circuit(circuit)

sim_probs = sim_result.subcircuits[0].probability_by_int

sample = np.random.choice(4, size=n_samples, p=sim_probs)

for count in sample:

probs[count] += 1 #Increment state counter

probs = probs/n_samples #Determine probabilities from sampling

term_probs += [probs] #Combine lists of probs of each term in Hamiltonian

This code would be replaced with calls to run the Jaqal circuit on the hardware when not using the emulator.

The following python code estimates the energy of a term after the appropriate rotations, discussed previously, have rotated the observables to each qubit’s *Z* basis. The following python functions, therefore, determine the expectation values of the diagonal operators. To determine the energy of a particular ansatz state, we define two functions:

# Calculate energy of one term of the Hamiltonian

# for one possible state

def term_energy(term, state, coefficient, prob):

parity = 1

for i in range(len(term)):

#Change parity if state is occupied

#and is acted on by a pauli operator

if term[i] != None and state[i] == ’1’:

parity = -1*parity

return coefficient*prob*parity

# Calculate energy of the molecule for a given value of theta

def make_calculate_energy(sample_noise=False):

def calculate_energy(theta):

energy = 0

#Convert tuple (from optimization) to float

probs = ansatz(theta[0], sample_noise)

for i in range(len(terms)): #For each term in the hamiltonian

for j in range(len(probs[0])): #For each possible state

term = terms[i]

#convert state to binary (# of qubits)

state = ’{0:02b}’.format(j)[::-1]

#binary must be inverted due to jaqalpaq convention

coefficient = cs[i].real

prob = probs[i][j]

energy += term_energy(term, state, coefficient, prob)

return energy

return calculate_energy

The first python function will calculate the energy of one particular basis state for one term in the Hamiltonian. The inputs to this function are the given Pauli term, the two-qubit basis state (in binary), the corresponding energy coefficient for the reduced Hamiltonian, and the probability of measuring that state. The code inside this function determines the parity of the state, which depends on whether or not each orbital is occupied and whether or not the qubit representing that orbital is acted upon by a Pauli operator. The function then returns the appropriate energy depending on that parity value.

The energy calculations for each term and basis state, as computed by the first function, are summed together in the second python function. This second function calculates the total energy of the molecule by running the ansatz circuit and measuring the energy of each of the terms in the Hamiltonian for all of the possible states created by the circuit.

### 4.2. $H_{2}$: Derivation of the UCCSD Operator

We still need to fill in the action of the ansatz circuit to prepare the state for measurement. If we encode the Unitary Coupled Cluster for single and double excitations (UCCSD) of $H_{2}$ from [28] with the Bravyi–Kitaev (BK) mapping, we obtain

(2) $$U\left(\theta \right)=\exp\left(i\frac{\theta }{8}\left[-X_{2}Y_{0}+Y_{2}X_{0}-X_{2}Z_{1}Y_{0}+Y_{2}Z_{1}X_{0}-Z_{3}X_{2}Y_{0}+Z_{3}Y_{2}X_{0}-Z_{3}X_{2}Z_{1}Y_{0}+Z_{3}Y_{2}Z_{1}X_{0}\right]\right).$$

As noted earlier, this operator acts with only the Pauli *I* and *Z* rotations on qubits 1 and 3, and thus it can be reduced to act on the qubits 0 and 2. When acting on the Bravyi–Kitaev Hartree–Fock state, further simplification (and a relabeling of the qubits 0 and 2 to 0 and 1) leads to the form:

(3) $$U\left(\theta \right)|01\rangle =\exp\left(-i\theta X_{1}Y_{0}\right)|01\rangle .$$

In the “standard quantum circuit gate basis” [6], this operator would be implemented with CNOT gates that entangle the qubits and measure their parity. However, CNOTs are not native gates to the QSCOUT trapped-ion qubit platform. An alternative is to replace those CNOT gates with a combination of rotations and Mølmer–Sørensen (MS) gates, as shown in  [33,34]. In the replacement circuit described in that reference, the qubits are entangled by a Mølmer–Sørensen gate that rotates by $+\frac{\pi }{2}$, followed by a parameterized *Z* rotation, followed by another Mølmer–Sørensen gate that rotates by $-\frac{\pi }{2}$, followed by a measurement.

This circuit can be written in JaqalPaq as follows:

# Prepare the Hartree Fock state

builder.gate(’prepare_all’)

builder.gate(’Px’, q[0])

# Apply the UCC Ansatz exp[-i*theta(X1 Y0)]

builder.gate(’MS’, q[1], q[0], 0, np.pi/2)

builder.gate(’Rz’, q[1], theta)

builder.gate(’MS’, q[1], q[0], 0, -np.pi/2)

In the preceding JaqalPaq code, we prepare the qubits in the $|00\rangle$ state and then act on the second qubit with an *X* rotation to produce the Hartree–Fock state $|01\rangle$. We then apply the UCCSD operator as described above using Mølmer–Sørensen gates. Note that Jaqal allows for a degree of freedom in choosing the axis angle of Mølmer–Sørensen gates, which can be used to simplify some circuits; however, in this case, we set the angle to 0. Our operator is parameterized using an argument theta input from our classical optimization loop. With the entire circuit prepared, we then minimize the energy via an external classical optimization routine. We used the minimization methods available in scipy.optimize [35]—specifically, the COBYLA algorithm. This code is shown in its entirety in Appendix A.

**Figure 2:**
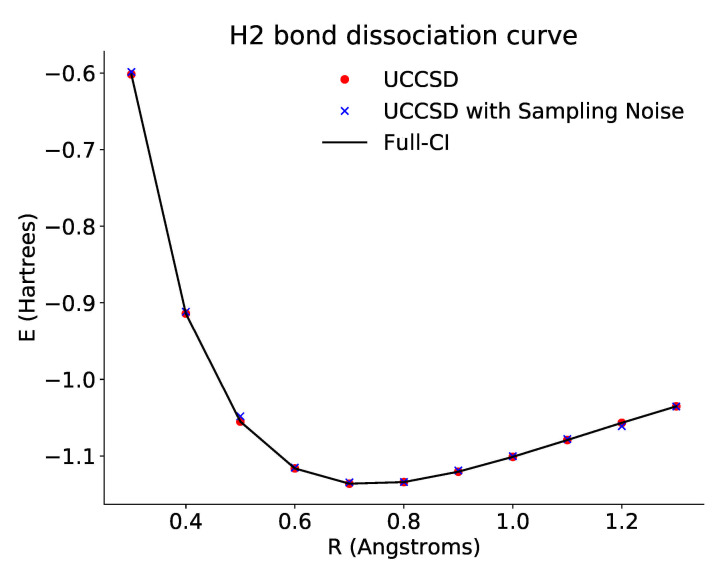
Plot of the dissociation curve of $H_{2}$ across different bond lengths. The black line is the theoretical full configuration interaction value for the ground state energy. The red points show the optimized energies derived from accessing the probabilities directly via the emulator. The blue points show the optimized energies derived from repeated sampling of the ansatz circuits. In both cases, the optimized results approach the theoretical curve.

The dissociation curve we computed using this JaqalPaq code is shown in Figure 2, using the built-in emulator capability in JaqalPaq. The data points fall exactly on the theoretical full configuration-interaction (Full-CI) curve for two reasons. First, the ansatz can realize the exact ground state for this small example. Second, the current version of the JaqalPaq emulator (a) models each QSCOUT operation as being either an error-free unitary transformation, state preparation, or measurement and (b) returns the exact final quantum state upon quantum circuit completion.

As such, these results do not suffer from fluctuations due to the finite numbers of samples. For larger examples, the ansatz state will, in general, not be able to realize the exact ground state energy. Simulation of the operation on QSCOUT with a finite number of samples will result in statistical fluctuations in the data as is also shown in Figure 2. For all sampling-based curves in this paper, we chose a baseline of 10,000 samples.

*$H_{2}$: Bond Dissociation Curve Results*

## 5. ${\mathrm{HeH}}^{+}$: Helium Hydride

Replacing one of the protons of the hydrogen molecule by an alpha particle gives ${\mathrm{HeH}}^{+}$. This example was realized experimentally in the original paper on VQE and has some interesting features, which we describe below [3].

### 5.1. ${HeH}^{+}$: Derivation of the Hamiltonian

Both $H_{2}$ and ${\mathrm{HeH}}^{+}$ have two electrons and two space orbitals; therefore, we will use a comparable Hamiltonian and ansatz as before. However, unlike $H_{2}$, the one body term of the ${\mathrm{HeH}}^{+}$ Hamiltonian is not symmetric under exchange of the nuclei. In the BK encoding, the Hamiltonian for ${\mathrm{HeH}}^{+}$ has 27 terms acting on four qubits. Using tapering techniques from [29] in OpenFermion [36], we may reduce this to nine terms acting on two qubits. As with our other molecules, we will create a Jaqal circuit for each of these terms, making the appropriate rotations at the end to measure the expectation value and calculate the energy. This is written in JaqalPaq as:

# Define terms and coefficients of our Hamiltonian

terms = []

cs = [] #Coefficients

for term in hamiltonian_bk.terms:

paulis = [None, None]

for pauli in term:

paulis[pauli[0]] = pauli[1]

terms += [paulis]

cs += [hamiltonian_bk.terms[term]]

for i in range(len(terms)):

builder = circuitbuilder.CircuitBuilder(

native_gates=normalize_native_gates(native_gates.NATIVE_GATES))

# Define constants +-pi/2

pi2 = builder.let(’pi2’, pi/2)

npi2 = builder.let(’npi2’, -pi/2)

# Create a qubit register

q = builder.register(’q’, 2)

# Define a hadamard macro

hadamard = circuitbuilder.SequentialBlockBuilder()

hadamard.gate(’Sy’, ’a’)

hadamard.gate(’Px’, ’a’)

builder.macro(’hadamard’, [’a’], hadamard)

#################

#Apply UCC Ansatz

#################

# Change basis for measurement depending on term

for j, qubit in enumerate(terms[i]):

if qubit == ’X’:

builder.gate(’hadamard’, (’array_item’, q, j)),

if qubit == ’Y’:

builder.gate(’Sxd’, (’array_item’, q, j)),

builder.gate(’measure_all’)

circuit = builder.build()

# Format results of simulation as a list of lists

sim_result = emulator.run_jaqal_circuit(circuit) #Run circuit on emulator

sim_probs = sim_result.subcircuits[0].probability_by_int #Extract probabilities

term_probs += [sim_probs] #Combine lists of probs of each term in Hamiltonian

Here, we begin again by creating nine Jaqal circuits. We previously derived our tapered Hamiltonian using PySCF [32] and OpenFermion [36] as shown in detail in Appendix B. By iterating over each of the terms in the Hamiltonian, we can separate out the Pauli operators and their corresponding coefficients for use in our algorithm. The rest of the code is the same as with $H_{2}$, where we iterate over each circuit and perform the necessary rotations to measure each term of our Hamiltonian. With this setup, we may then calculate the energy using the same functions as with $H_{2}$—this time using the terms and coefficients calculated above instead of putting them in by hand.

### 5.2. ${HeH}^{+}$: Derivation of the UCCSD Operator

For ${\mathrm{HeH}}^{+}$, the UCCSD operator will be the same as with $H_{2}$, as we are again interested in only the excitations between the two space orbitals of the molecule. However, due to a change in the ordering of qubits in OpenFermion we are now acting on the state $|11\rangle$:

(4) $$U\left(\theta \right)|11\rangle =\exp\left(-i\theta X_{0}Y_{1}\right)|11\rangle ,$$

which can be implemented in JaqalPaq as shown in the previous section:

# Prepare the Hartree Fock state

builder.gate(’prepare_all’)

builder.gate(’Px’, q[0])

builder.gate(’Px’, q[1])

# Apply the UCC Ansatz exp[-i*theta(X1 Y0)]

builder.gate(’MS’, q[1], q[0], 0, pi2)

builder.gate(’Rz’, q[1], theta)

builder.gate(’MS’, q[1], q[0], 0, npi2)

**Figure 3:**
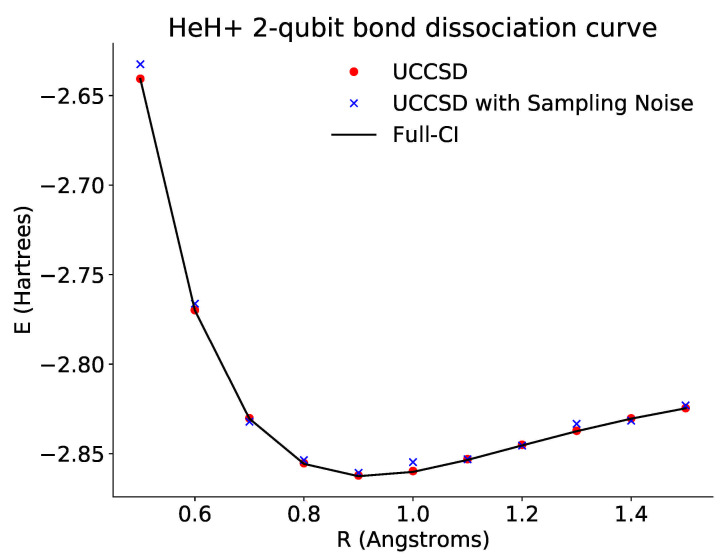
Plot of the dissociation curve of ${\mathrm{HeH}}^{+}$ for a tapered two-qubit Hamiltonian across different bond lengths. The black line is the theoretical full configuration interaction value for the ground state energy. The red points show the optimized energies derived from accessing the probabilities directly via the emulator. The blue points show the optimized energies derived from repeated sampling of the ansatz circuits. In both cases, the optimized results approach the theoretical curve.

As before, we prepare the HF state of our molecule and then apply our ansatz before rotating by our parameter “theta”. We may then classically optimize this parameter using scipy to find the ground state energy. These results are shown in Figure 3.

   *${\mathrm{HeH}}^{+}$: Bond Dissociation Curve Results*

## 6. LiH: Lithium Hydride

Our last example is a more complicated system, lithium hydride. The VQE algorithm for this system was previously experimentally realized using an ion trap in [28].

### 6.1. LiH: Derivation of the Hamiltonian

While $H_{2}$ and ${\mathrm{HeH}}^{+}$ have two relevant space orbitals in a minimal basis, LiH has six, meaning there are twelve spin orbitals to consider. In addition, there are now four electrons instead of two, dramatically increasing the number of orbital excitations that need to be taken into account. We again follow the work of Hempel et al. [28], choosing this time to use the STO-6G basis as they do for a more straightforward comparison. A naive encoding of the Hamiltonian using the Bravyi–Kitaev mapping would require 12 qubits, which is too many for the QSCOUT system. However, since we are only trying to approximate the ground state energy rather than calculate it exactly, we need not consider some of the molecular orbitals. By reducing the active space of our problem as shown in [28], we go from six orbitals and 12 qubits to three orbitals and six qubits.

Moreover, as we need only to consider transitions between the first and second orbitals and first and third orbitals, we simplify our UCCSD operator as well. This operator only acts non-trivially on three of the six qubits, meaning we may again reduce our Hamiltonian using the tapering techniques from [29]. This reduction is shown in more detail in Appendix C. In the Bravyi–Kitaev encoding, we obtain a Hamiltonian that acts on three qubits with a corresponding Hartree–Fock state $|\Psi_{HF}\rangle =|111\rangle$:

(5) $$H_{BK}=c_{0}I+c_{1}Z_{0}+c_{2}Z_{1}+c_{3}Z_{2}+c_{5}Z_{0}Z_{2}+c_{6}Z_{1}Z_{2}+c_{7}X_{0}X_{1}+c_{8}Y_{0}Y_{1}+c_{9}X_{0}X_{2}+c_{10}Y_{0}Y_{2}+c_{11}X_{1}X_{2}+c_{12}Y_{1}Y_{2}$$

This is expressed in JaqalPaq as follows:

# Reduce the BK Hamiltonian for LiH

terms = []

cs = []

red_hamiltonian_bk = QubitOperator()

result = reduce_hamiltonian(hamiltonian_bk)

for i in range(len(result)): #Separate out term and coeffs again after combining like terms

terms += [result[i][0]]

cs += [result[i][1]]

string = ’’

for j, pauli in enumerate(result[i][0]):

if pauli != None:

string += str(pauli)+str(j)+’ ’

red_hamiltonian_bk += result[i][1]*QubitOperator(string)

for i in range(len(terms)):

builder = circuitbuilder.CircuitBuilder(

native_gates=normalize_native_gates(native_gates.NATIVE_GATES))

# Define constants +-pi/2

pi2 = builder.let(’pi2’, pi/2)

npi2 = builder.let(’npi2’, -pi/2)

# Create a qubit register

q = builder.register(’q’, 3)

# Define a hadamard macro

hadamard = circuitbuilder.SequentialBlockBuilder()

hadamard.gate(’Sy’, ’a’)

hadamard.gate(’Px’, ’a’)

builder.macro(’hadamard’, [’a’], hadamard)

#################

#Apply UCCSD Ansatz

#################

# Change basis for measurement depending on term

for j, qubit in enumerate(terms[i]):

if qubit == ’X’:

builder.gate(’hadamard’, (’array_item’, q, j)),

if qubit == ’Y’:

builder.gate(’Sxd’, (’array_item’, q, j)),

builder.gate(’measure_all’)

circuit = builder.build()

# Format results of simulation as a list of lists

sim_result = emulator.run_jaqal_circuit(circuit) #Run circuit on emulator

sim_probs = sim_result.subcircuits[0].probability_by_int #Extract probabilities

term_probs += [sim_probs] #Combine lists of probs of each term in Hamiltonian

This code is of the same form and function as the code for $H_{2}$ and ${\mathrm{HeH}}^{+}$, with the main difference being how the terms and coefficients of the reduced Hamiltonian are calculated. A new function, defined in Appendix C, is called to transform the six-qubit Hamiltonian into a three-qubit version in the algorithm.

### 6.2. LiH: Derivation of the UCCSD Operator

As was mentioned in the previous section, we are interested only in the excitations among the first, second, and third orbitals. As with $H_{2}$, the UCCSD operator will be made from excitation operators; however, in this case, there will be two such operators leading to a two-parameter ansatz. In the Bravyi–Kitaev encoding, each of these operators has many Pauli terms as shown in Equation (2), corresponding to many gate operations. However, we may limit these expressions to only the first term in that expression without decreasing the accuracy of our calculation beyond acceptable bounds. Each of these operators may then be written as:

(6) $$U\left(\alpha \right)=\exp\left(-i\alpha X_{2}Y_{4}\right).$$

(7) $$U\left(\beta \right)=\exp\left(-i\beta X_{2}Y_{6}\right).$$

We see that these operators only ever act on qubits 2, 4, and 6, meaning that we may reduce our circuit and Hamiltonian to only act on three qubits in total, as discussed in the previous section. As was the case with $H_{2}$, these operators may be implemented using Mølmer–Sørensen gates and parameterized Z rotations in JaqalPaq:

# Prepare the Hartree Fock state

builder.gate(’prepare_all’)

builder.gate(’Px’, q[0])

builder.gate(’Px’, q[1])

builder.gate(’Px’, q[2])

# Apply the UCC Ansatz exp[-i*theta(X1 Y0)]

builder.gate(’MS’, q[1], q[0], 0, pi2)

builder.gate(’Rz’, q[0], alpha)

builder.gate(’MS’, q[1], q[0], 0, npi2)

builder.gate(’MS’, q[2], q[0], 0, pi2)

builder.gate(’Rz’, q[0], beta)

builder.gate(’MS’, q[2], q[0], 0, npi2)

**Figure 4:**
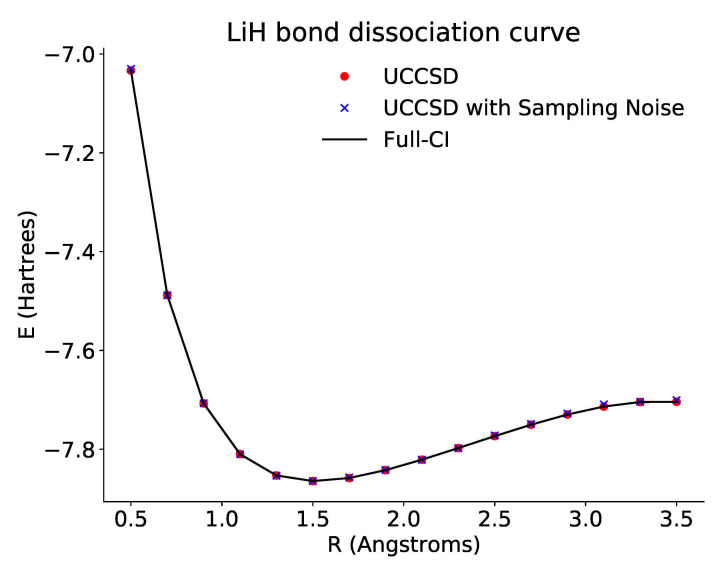
Plot of the dissociation curve of LiH for a tapered three-qubit Hamiltonian across different bond lengths. The black line is the theoretical full configuration interaction value for the ground state energy. Here, the exact energy is calculated based on the reduced three-qubit Hamiltonian. The red points show the optimized energies derived from accessing the probabilities directly via the emulator. The blue points show the optimized energies derived from repeated sampling of the ansatz circuits. In both cases, the optimized results approach the theoretical curve.

Here, we prepare the initial state with X gates as before, and then act on it with two operators, one for each parameter. As before, the resulting state is then rotated to the proper basis before measurement. The bond dissociation curve derived from this minimization is shown in Figure 4.

*LiH: Bond Dissociation Curve Results*

## 7. Molecules on Future Hardware

The current QSCOUT system has three qubits at its disposal, which limits the kinds of VQE experiments that can be run on the hardware. In this section, we detail future experiments that could be carried out on more qubits for QSCOUT 2.0 and beyond.

### ${HeH}^{+}$: Variant for a Four-Qubit Computer

An alternative solution for ${\mathrm{HeH}}^{+}$ is to use the untapered Hamiltonian for ${\mathrm{HeH}}^{+}$ that contains 27 terms acting on four qubits. This implementation is infeasible on current hardware, but it remains useful as an exercise. For this more general case, the only difference comes in the size of the qubit register:

# Create a qubit register

q = builder.register(’q’, 4)

and in the action of the UCCSD operator:

(8) $$UCC\left(\theta \right)|0011\rangle =\exp\left(-i\theta X_{0}X_{1}X_{2}Y_{3}\right)|0011\rangle ,$$

which can be implemented in Jaqal by using Mølmer–Sørensen gates as shown in Maslov et al. [34]:

# Define a CNOT macro

CNOT = circuitbuilder.SequentialBlockBuilder()

CNOT.gate(’Sy’, ’control’)

CNOT.gate(’MS’, ’control’, ’target’, 0, np.pi/2)

Sxd_block = CNOT.block(parallel=True)

Sxd_block.gate(’Sxd’, ’control’)

Sxd_block.gate(’Sxd’, ’target’)

CNOT.gate(’Syd’, ’control’)

builder.macro(’CNOT’, [’control’, ’target’], CNOT)

# Prepare the Hartree Fock state

builder.gate(’prepare_all’)

builder.gate(’Px’, q[0])

builder.gate(’Px’, q[1])

# Apply the UCC Ansatz exp[-i*theta(X1 Y0)]

builder.gate(’hadamard’, q[0])

builder.gate(’hadamard’, q[1])

builder.gate(’hadamard’, q[2])

builder.gate(’Sxd’, q[3])

builder.gate(’CNOT’, q[0], q[1])

builder.gate(’CNOT’, q[1], q[2])

builder.gate(’CNOT’, q[2], q[3])

builder.gate(’Rz’, q[3], theta)

builder.gate(’CNOT’, q[2], q[3])

builder.gate(’CNOT’, q[1], q[2])

builder.gate(’CNOT’, q[0], q[1])

builder.gate(’hadamard’, q[0])

builder.gate(’hadamard’, q[1])

builder.gate(’hadamard’, q[2])

builder.gate(’Sx’, q[3])

In this code, we define a `CNOT’ macro as a combination of rotations and a Mølmer–Sørensen gate using nested sequential and parallel blocks of Jaqal code. This macro can then be used in the ansatz circuit to entangle the four qubits together to prepare the ansatz state.

**Figure 5:**
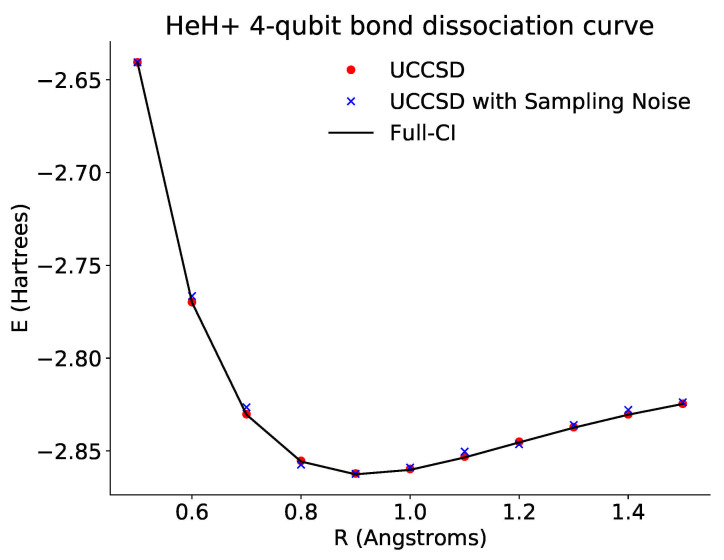
Plot of the dissociation curve of ${\mathrm{HeH}}^{+}$ for the full four-qubit Hamiltonian across different bond lengths. The black line is the theoretical full configuration interaction value for the ground state energy. The red points show the optimized energies derived from accessing the probabilities directly via the emulator. The blue points show the optimized energies derived from repeated sampling of the ansatz circuits. In both cases, the optimized results approach the theoretical curve.

Here, we plotted the bond dissociation curve for ${\mathrm{HeH}}^{+}$ for four qubits (Figure 5).

## 8. Discussion and Conclusions

We showed the process of computing the ground state energy of three different small molecules: $H_{2}$, ${\mathrm{HeH}}^{+}$, and LiH. We began with the derivation of the molecules’ respective Hamiltonians through quantum chemistry packages, such as PySCF [32] and OpenFermion [36]. Then, for each molecule, we iteratively prepared and measured a VQE ansatz state using the tools that JaqalPaq and its emulator provide, including an exact method and sampling based method. Lastly, we plotted the results of these simulations as bond dissociation curves, comparing our optimized values to the theoretical Full-CI values for each molecule. We hope that by presenting and explaining these example algorithms for $H_{2}$, ${\mathrm{HeH}}^{+}$, and LiH that beginning JaqalPaq programmers will have a starting point for developing their own variational quantum algorithms using Jaqal.

## Figures and Tables

**Table 1:** Molecules for which we present JaqalPaq exemplars. The numbers of orbitals and qubits used have been reduced using the techniques described in the corresponding sections.

Molecule	Protons	Electrons	Orbitals	Hamiltonian	Qubits	Basis	Section
				Terms			
$H_{2}$	2	2	2	6	2	STO-3G	Section 4
${\mathrm{HeH}}^{+}$	3	2	2	9	2	STO-3G	Section 5
LiH	4	4	3	13	3	STO-6G	Section 6

## Data Availability

Code available at https://gitlab.com/jaqal (accessed on 23 May 2021).

## Appendix A. H_2_ Code

# Imports for QSCOUT

import jaqalpaq

from jaqalpaq.core import circuitbuilder

from jaqalpaq.core.circuit import normalize_native_gates

from jaqalpaq import emulator

from qscout.v1 import native_gates

# Imports for basic mathematical functionality

from math import pi

import numpy as np

# Imports for OpenFermion(-PySCF)

import openfermion as of

from openfermion.chem import MolecularData

from openfermionpyscf import run_pyscf

# Import for VQE optimizer

from scipy import optimize

def ansatz(theta, sample_noise=False):

    term_probs = []

    for i in range(len(terms)):

        builder = circuitbuilder.CircuitBuilder(

                       native_gates=normalize_native_gates(native_gates.NATIVE_GATES))

        # Create a qubit register

        q = builder.register(’q’, 2)

        # Define a hadamard macro

        hadamard = circuitbuilder.SequentialBlockBuilder()

        hadamard.gate(’Sy’, ’a’)

        hadamard.gate(’Px’, ’a’)

        builder.macro(’hadamard’, [’a’], hadamard)

        # Prepare the Hartree Fock state

        builder.gate(’prepare_all’)

        builder.gate(’Px’, q[0])

        # Apply the UCC Ansatz exp[-i*theta(X1 Y0)]

        builder.gate(’MS’, q[1], q[0], 0, np.pi/2)

        builder.gate(’Rz’, q[1], theta)

        builder.gate(’MS’, q[1], q[0], 0, -np.pi/2)

        # Change basis for measurement depending on term

        for j, qubit in enumerate(terms[i]):

            if qubit == ’X’:

                builder.gate(’hadamard’, (’array_item’, q, j)),

            if qubit == ’Y’:

                builder.gate(’Sxd’, (’array_item’, q, j)),

        builder.gate(’measure_all’)

        circuit = builder.build()

        # Format results of simulation as a list of lists

        sim_result = emulator.run_jaqal_circuit(circuit)

        sim_probs = sim_result.subcircuits[0].probability_by_int

        if sample_noise: #Sample circuits to determine probs

            probs = np.zeros(4) # Number of possible states

            for k in range(n_samples):

                sample = np.random.choice(4, p=sim_probs)

                probs[sample] += 1 #Increment state counter

            probs = probs/n_samples #Determine probabilities from sampling

            term_probs += [probs] #Combine lists of probs of each term in Hamiltonian

        else: #Exact solution without sampling

            term_probs += [sim_probs]

    return term_probs

# Calculate energy of one term of the Hamiltonian for one possible state

def term_energy(term, state, coefficient, prob):

    parity = 1

    for i in range(len(term)):

        #Change parity if state is occupied and is acted on by a pauli operator

        if term[i] != None and state[i] == ’1’:

            parity = -1*parity

    return coefficient*prob*parity

# Calculate energy of the molecule for a given value of theta

def make_calculate_energy(sample_noise=False):

    def calculate_energy(theta):

        energy = 0

        probs = ansatz(theta[0], sample_noise) #Convert tuple (from optimization) to float

        for i in range(len(terms)): #For each term in the hamiltonian

            for j in range(len(probs[0])): #For each possible state

                term = terms[i]

                state = ’{0:02b}’.format(j)[::-1] #convert state to binary (# of qubits)

                #binary must be inverted due to jaqalpaq convention

                coefficient = cs[i].real

                prob = probs[i][j]

                energy += term_energy(term, state, coefficient, prob)

        return energy

    return calculate_energy

# Set the basis set, spin, and charge of the H2 molecule

basis = ’sto-3g’

multiplicity = 1

charge = 0

# Set calculation parameters

run_scf = 1

run_fci = 1

delete_input = True

delete_output = False

optimized_energies = [[], []]

exact_energies = []

# Loop over bond lengths from 0.3 to 1.3 angstroms

sample_noise = False

n_samples = 10000 # Sample circuit

n_pts = 11 # Number of points

bond_lengths = np.linspace(0.3,1.3,n_pts)

for diatomic_bond_length in bond_lengths:

    # Generate molecule at some bond length

    geometry = [(’H’, (0., 0., 0.)), (’H’, (0., 0., diatomic_bond_length))]

    molecule = MolecularData(

        geometry, basis, multiplicity, charge,

        description=str(round(diatomic_bond_length, 2)),

        filename=’./H2_sto-3g_single_dissociation’)

    # Run pyscf to generate new molecular data for sto-3g H2

    molecule = run_pyscf(molecule,

                     run_scf=run_scf,

                     run_fci=run_fci,

                     verbose=False)

    # Get the fermionic Hamiltonian for H2 and map it into qubits using the BK encoding

    hamiltonian = molecule.get_molecular_hamiltonian()

    hamiltonian_ferm = of.get_fermion_operator(hamiltonian)

    hamiltonian_bk = of.bravyi_kitaev(hamiltonian_ferm)

    # Define Pauli strings that appear in the reduced two-qubit Hamiltonian

    #          q0 ,  q1

    terms = [[None, None], [’Z’, None], [None, ’Z’], [’Z’, ’Z’], [’X’, ’X’], [’Y’, ’Y’]]

    # Calculate effective coefficients for the reduced two-qubit Hamiltonian

    # Derivation follows arXiv:1803.10238v2 appendix A-2

    fs = hamiltonian_bk.terms #Old coefficients from OpenFermion Hamiltonian

    c0 = (fs[()] + fs[(1, ’Z’),] + fs[(1, ’Z’), (3, ’Z’),]).real

    c1 = (fs[(0, ’Z’),] + fs[(0, ’Z’), (1, ’Z’),]).real

    c2 = (fs[(2, ’Z’),] + fs[(1, ’Z’), (2, ’Z’), (3, ’Z’),]).real

    c3 = (fs[(0, ’Z’), (2, ’Z’),] + fs[(0, ’Z’), (1, ’Z’), (2, ’Z’),] + fs[(0, ’Z’), (2, ’Z’),

        (3, ’Z’),] + fs[(0, ’Z’), (1, ’Z’), (2, ’Z’), (3, ’Z’)]).real

    c4 = (fs[(0,’X’), (1, ’Z’), (2, ’X’),] + fs[(0, ’X’), (1, ’Z’), (2, ’X’), (3, ’Z’),]).real

    c5 = (fs[(0, ’Y’), (1, ’Z’), (2, ’Y’),] + fs[(0, ’Y’), (1, ’Z’), (2, ’Y’), (3, ’Z’),]).real

    cs = [c0, c1, c2, c3, c4, c5] #New coefficients are linear combinations of old coefficients

    # Minimize the expectation value of the energy using a classical optimizer (COBYLA)

    exact_energies.append(molecule.fci_energy)

    print("R={}\t Exact Energy: {}".format(str(round(diatomic_bond_length, 2)), molecule.fci_energy))

    for i in range(2):

        result = optimize.minimize(fun=make_calculate_energy(sample_noise=i), x0=[0.01], method="COBYLA")

        optimized_energies[i].append(result.fun)

        print("R={}\t Optimized Energy: {}\t Sampling Noise: {}".format(

                                   str(round(diatomic_bond_length, 2)), result.fun, bool(i)))

    print("\n")

import matplotlib

import matplotlib.pyplot as pyplot

# Plot the various energies for different bond lengths

fig = pyplot.figure(figsize=(15,10))

pyplot.rcParams[’font.size’]=18

bkcolor = ’#ffffff’

ax = fig.add_subplot(1, 1, 1)

pyplot.subplots_adjust(left=.2)

ax.set_xlabel(’R (Angstroms)’)

ax.set_ylabel(r’E (Hartrees)’)

ax.set_title(r’H2 bond dissociation curve’)

ax.spines[’right’].set_visible(False)

ax.spines[’top’].set_visible(False)

bond_lengths = [float(x) for x in bond_lengths]

ax.plot(bond_lengths, optimized_energies[0], ’o’, label=’UCCSD’, color=’red’)

ax.plot(bond_lengths, optimized_energies[1], ’x’, label=’UCCSD with Sampling Noise’, color=’blue’)

ax.plot(bond_lengths, exact_energies, ’-’, label=’Full-CI’, color=’black’)

ax.legend(frameon=False)

pyplot.show()

## Appendix B. HeH^+^ Code

# Imports for QSCOUT

import jaqalpaq

from jaqalpaq.core import circuitbuilder

from jaqalpaq.core.circuit import normalize_native_gates

from jaqalpaq import emulator

from qscout.v1 import native_gates

# Imports for basic mathematical functionality

from math import pi

import numpy as np

# Imports for OpenFermion(-PySCF)

import openfermion as of

from openfermion.chem import MolecularData

from openfermionpyscf import run_pyscf

# Import for VQE optimizer

from scipy import optimize

def ansatz(theta, sample_noise=False):

    term_probs = []

    for i in range(len(terms)):

        builder = circuitbuilder.CircuitBuilder(

                       native_gates=normalize_native_gates(native_gates.NATIVE_GATES))

        # Create a qubit register

        q = builder.register(’q’, 2)

        # Define a hadamard macro

        hadamard = circuitbuilder.SequentialBlockBuilder()

        hadamard.gate(’Sy’, ’a’)

        hadamard.gate(’Px’, ’a’)

        builder.macro(’hadamard’, [’a’], hadamard)

        # Prepare the Hartree Fock state

        builder.gate(’prepare_all’)

        builder.gate(’Px’, q[0])

        builder.gate(’Px’, q[1])

        # Apply the UCC Ansatz exp[-i*theta(X1 Y0)]

        builder.gate(’MS’, q[1], q[0], 0, np.pi/2)

        builder.gate(’Rz’, q[1], theta)

        builder.gate(’MS’, q[1], q[0], 0, -np.pi/2)

        # Change basis for measurement depending on term

        for j, qubit in enumerate(terms[i]):

            if qubit == ’X’:

                builder.gate(’hadamard’, (’array_item’, q, j)),

            if qubit == ’Y’:

                builder.gate(’Sxd’, (’array_item’, q, j)),

        builder.gate(’measure_all’)

        circuit = builder.build()

        # Format results of simulation as a list of lists

        sim_result = emulator.run_jaqal_circuit(circuit)

        sim_probs = sim_result.subcircuits[0].probability_by_int

        if sample_noise: #Sample circuits to determine probs

            probs = np.zeros(4) # Number of possible states

            for k in range(n_samples):

                sample = np.random.choice(4, p=sim_probs)

                probs[sample] += 1 #Increment state counter

            probs = probs/n_samples #Determine probabilities from sampling

            term_probs += [probs] #Combine lists of probs of each term in Hamiltonian

        else: #Exact solution without sampling

            term_probs += [sim_probs]

    return term_probs

# Calculate energy of one term of the Hamiltonian for one possible state

def term_energy(term, state, coefficient, prob):

    parity = 1

    for i in range(len(term)):

        #Change parity if state is occupied and is acted on by a pauli operator

        if term[i] != None and state[i] == ’1’:

            parity = -1*parity

    return coefficient*prob*parity

# Calculate energy of the molecule for a given value of theta

def make_calculate_energy(sample_noise=False):

    def calculate_energy(theta):

        energy = 0

        probs = ansatz(theta[0], sample_noise) #Convert tuple (from optimization) to float

        for i in range(len(terms)): #For each term in the hamiltonian

            for j in range(len(probs[0])): #For each possible state

                term = terms[i]

                state = ’{0:02b}’.format(j)[::-1] #convert state to binary (# of qubits)

                #binary must be inverted due to jaqalpaq convention

                coefficient = cs[i].real

                prob = probs[i][j]

                energy += term_energy(term, state, coefficient, prob)

        return energy

    return calculate_energy

# Set the basis set, spin, and charge of the HeH+ molecule

basis = ’sto-3g’

multiplicity = 1

charge = 1 #Charge is 1 for HeH+

# Set calculation parameters

run_scf = 1

run_fci = 1

delete_input = True

delete_output = False

optimized_energies = [[], []]

exact_energies = []

# Loop over bond lengths from 0.5 to 1.5 angstroms

sample_noise = False

n_samples = 10000

n_pts = 11 # Number of points

bond_lengths = np.linspace(0.5,1.5,n_pts)

for diatomic_bond_length in bond_lengths:

    # Generate molecule at some bond length

    geometry = [(’He’, (0., 0., 0.)), (’H’, (0., 0., diatomic_bond_length))]

    molecule = MolecularData(

        geometry, basis, multiplicity, charge,

        description=str(round(diatomic_bond_length, 2)),

        filename=’./HeH+_2_sto-3g_single_dissociation’)

    # Run pyscf to generate new molecular data for sto-3g HeH+

    molecule = run_pyscf(molecule,

                     run_scf=run_scf,

                     run_fci=run_fci,

                     verbose=False)

    # Get the fermionic Hamiltonian for HeH+ and map it using the BK encoding

    hamiltonian = molecule.get_molecular_hamiltonian()

    hamiltonian_ferm = of.get_fermion_operator(hamiltonian)

    hamiltonian_bk = of.symmetry_conserving_bravyi_kitaev(

                                hamiltonian_ferm, active_orbitals=4, active_fermions=2)

    # Define terms and coefficients of our Hamiltonian

    terms = []

    cs = [] #Coefficients

    for term in hamiltonian_bk.terms:

        paulis = [None, None]

        for pauli in term:

            paulis[pauli[0]] = pauli[1]

        terms += [paulis]

        cs += [hamiltonian_bk.terms[term]]

    # Minimize the expectation value of the energy using a classical optimizer (COBYLA)

    exact_energies.append(molecule.fci_energy)

    print("R={}\t Exact Energy: {}".format(

                             str(round(diatomic_bond_length, 2)), molecule.fci_energy))

    for i in range(2):

        result = optimize.minimize(fun=make_calculate_energy(sample_noise=i), x0=[0.01], method="COBYLA")

        optimized_energies[i].append(result.fun)

        print("R={}\t Optimized Energy: {}\t Sampling Noise: {}".format(

                                str(round(diatomic_bond_length, 2)), result.fun, bool(i)))

    print("\n")

import matplotlib

import matplotlib.pyplot as pyplot

# Plot the various energies for different bond lengths

fig = pyplot.figure(figsize=(15,10))

pyplot.rcParams[’font.size’]=18

bkcolor = ’#ffffff’

ax = fig.add_subplot(1, 1, 1)

pyplot.subplots_adjust(left=.2)

ax.set_xlabel(’R (Angstroms)’)

ax.set_ylabel(r’E (Hartrees)’)

ax.set_title(r’HeH+ 2-qubit bond dissociation curve’)

ax.spines[’right’].set_visible(False)

ax.spines[’top’].set_visible(False)

bond_lengths = [float(x) for x in bond_lengths]

ax.plot(bond_lengths, optimized_energies[0], ’o’, label=’UCCSD’, color=’red’)

ax.plot(bond_lengths, optimized_energies[1], ’x’, label=’UCCSD with Sampling Noise’, color=’blue’)

ax.plot(bond_lengths, exact_energies, ’-’, label=’Full-CI’, color=’black’)

ax.legend(frameon=False)

pyplot.show()

## Appendix C. LiH Code

# Imports for QSCOUT

import jaqalpaq

from jaqalpaq.core import circuitbuilder

from jaqalpaq.core.circuit import normalize_native_gates

from jaqalpaq import emulator

from qscout.v1 import native_gates

# Imports for basic mathematical functionality

from math import pi

import numpy as np

# Imports for OpenFermion(-PySCF)

import openfermion as of

from openfermion.chem import MolecularData

from openfermionpyscf import run_pyscf

from openfermion.ops import QubitOperator

from openfermion.utils import eigenspectrum

# Import for VQE optimizer

from scipy import optimize

def ansatz(alpha, beta, sample_noise=False):

    term_probs = []

    for i in range(len(terms)):

        builder = circuitbuilder.CircuitBuilder(native_gates=

            normalize_native_gates(native_gates.NATIVE_GATES))

        # Create a qubit register

        q = builder.register(’q’, 3)

        # Define a hadamard macro

        hadamard = circuitbuilder.SequentialBlockBuilder()

        hadamard.gate(’Sy’, ’a’)

        hadamard.gate(’Px’, ’a’)

        builder.macro(’hadamard’, [’a’], hadamard)

        # Prepare the Hartree Fock state

        builder.gate(’prepare_all’)

        builder.gate(’Px’, q[0])

        builder.gate(’Px’, q[1])

        builder.gate(’Px’, q[2])

        # Apply the UCC Ansatz exp[-i*theta(X1 Y0)]

        builder.gate(’MS’, q[0], q[1], 0, np.pi/2)

        builder.gate(’Rz’, q[0], alpha)

        builder.gate(’MS’, q[0], q[1], 0, -np.pi/2)

        builder.gate(’MS’, q[0], q[2], 0, np.pi/2)

        builder.gate(’Rz’, q[0], beta)

        builder.gate(’MS’, q[0], q[2], 0, -np.pi/2)

        # Change basis for measurement depending on term

        for j, qubit in enumerate(terms[i]):

            if qubit == ’X’:

                builder.gate(’hadamard’, (’array_item’, q, j)),

            if qubit == ’Y’:

                builder.gate(’Sxd’, (’array_item’, q, j)),

        builder.gate(’measure_all’)

        circuit = builder.build()

        # Format results of simulation as a list of lists

        sim_result = emulator.run_jaqal_circuit(circuit)

        sim_probs = sim_result.subcircuits[0].probability_by_int

        if sample_noise: #Sample circuits to determine probs

            probs = np.zeros(8) # Number of possible states

            for k in range(n_samples):

                sample = np.random.choice(8, p=sim_probs)

                probs[sample] += 1 #Increment state counter

            probs = probs/n_samples #Determine probabilities from sampling

            term_probs += [probs] #Combine lists of probs of each term in Hamiltonian

        else: #Exact solution without sampling

            term_probs += [sim_probs]

    return term_probs

# Calculate energy of one term of the Hamiltonian for one possible state

def term_energy(term, state, coefficient, prob):

    parity = 1

    for i in range(len(term)):

        #Change parity if state is occupied and is acted on by a pauli operator

        if term[i] != None and state[i] == ’1’:

            parity = -1*parity

    return coefficient*prob*parity

# Calculate energy of the molecule for a given value of theta

def make_calculate_energy(sample_noise=False):

    def calculate_energy(params):

        energy = 0

        probs = ansatz(params[0], params[1], sample_noise) #Convert tuple to float for circuit

        for i in range(len(terms)): #For each term in the hamiltonian

            for j in range(len(probs[0])): #For each possible state

                term = terms[i]

                state = ’{0:03b}’.format(j)[::-1] #Formatted by number of qubits

                #binary must be inverted due to jaqalpaq convention

                coefficient = cs[i].real

                prob = probs[i][j]

                energy += term_energy(term, state, coefficient, prob)

        return energy

    return calculate_energy

# Further reduce the Hamiltonian to 3 qubits

def reduce_hamiltonian(hamiltonian):

    terms = []

    cs = []

    for term in hamiltonian.terms:

        paulis = [None, None, None]

        ignore_term = False

        for pauli in term:

            #HF state |000001> {indices 7,6,5,4,3,2 -> 5,4,3,2,1,0}

            #Ansatz does not act on odd indices so they may be classically evaluated

            if pauli[0]%2==1 and (pauli[1]==’X’ or pauli[1]==’Y’): #expectation value is 0

                ignore_term = True

            #Ansatz does act on even indices so they are left alone and relabled

            elif pauli[0]%2==0:

                paulis[pauli[0]//2] = pauli[1]

        if not ignore_term: #For terms that remain, separate out paulis and coefficients

            terms += [paulis]

            cs += [hamiltonian.terms[term]]

    # Combine like terms in the Hamiltonian

    d = {}

    for i in range(len(terms)):

        if repr(terms[i]) in d: #If term is present...

            d[repr(terms[i])][1] += cs[i] #Sum coefficients for simplified pauli terms

        else: #If term isn’t present...

            d[repr(terms[i])] = [terms[i], cs[i]] #Simply append it

    return(list(d.values()))

# Set the basis set, spin, and charge of the LiH molecule

basis = ’sto-6g’

multiplicity = 1

charge = 0

# Set calculation parameters

run_scf = 1

run_fci = 1

delete_input = True

delete_output = False

optimized_energies = [[], []]

exact_energies = []

hf_energies = []

# Loop over bond lengths from 1.0 to 2.0 angstroms

sample_noise=False

n_samples = 10000 # Sample circuit

n_pts = 11 #Number of points

bond_lengths = np.linspace(1.0,2.0,n_pts)

for diatomic_bond_length in bond_lengths:

    # Generate molecule at some bond length

    geometry = [(’Li’, (0., 0., 0.)), (’H’, (0., 0., diatomic_bond_length))]

    molecule = MolecularData(

        geometry, basis, multiplicity, charge,

        description=str(round(diatomic_bond_length, 2)),

        filename=’./LiH_sto-6g_single_dissociation’)

    # Run pyscf

    molecule = run_pyscf(molecule,

                     run_scf=run_scf,

                     run_fci=run_fci,

                     verbose=False)

    # Get the fermionic Hamiltonian for LiH and map it using the BK encoding

    # Reduce active space to orbitals 1, 2, and 3

    hamiltonian = molecule.get_molecular_hamiltonian(occupied_indices=[0], active_indices=[1,2,3])

    hamiltonian_ferm = of.get_fermion_operator(hamiltonian)

    hamiltonian_bk = of.bravyi_kitaev(hamiltonian_ferm)

    # Reduce the BK Hamiltonian for LiH

    terms = []

    cs = []

    red_hamiltonian_bk = QubitOperator()

    result = reduce_hamiltonian(hamiltonian_bk)

    for i in range(len(result)): #Separate out term and coeffs again after combining like terms

        terms += [result[i][0]]

        cs += [result[i][1]]

        string = ’’

        for j, pauli in enumerate(result[i][0]):

            if pauli != None:

                string += str(pauli)+str(j)+’ ’

        red_hamiltonian_bk += result[i][1]*QubitOperator(string)

    # Minimize the expectation value of the energy using a classical optimizer (COBYLA)

    exact_energies.append(eigenspectrum(red_hamiltonian_bk)[0])

    print("R={}\t Exact Energy: {}".format(

                      str(round(diatomic_bond_length, 2)),eigenspectrum(red_hamiltonian_bk)[0]))

    for i in range(2):

        result = optimize.minimize(

                     fun=make_calculate_energy(sample_noise=i), x0=[0.01, 0.01], method="COBYLA")

        optimized_energies[i].append(result.fun)

        print("R={}\t Optimized Energy: {}\t Sampling Noise: {}".format(

                                  str(round(diatomic_bond_length, 2)), result.fun, bool(i)))

    print("\n")

import matplotlib

import matplotlib.pyplot as pyplot

# Plot the various energies for different bond lengths

fig = pyplot.figure(figsize=(15,10))

pyplot.rcParams[’font.size’]=18

bkcolor = ’#ffffff’

ax = fig.add_subplot(1, 1, 1)

pyplot.subplots_adjust(left=.2)

ax.set_xlabel(’R (Angstroms)’)

ax.set_ylabel(r’E (Hartrees)’)

ax.set_title(r’LiH bond dissociation curve’)

ax.spines[’right’].set_visible(False)

ax.spines[’top’].set_visible(False)

bond_lengths = [float(x) for x in bond_lengths]

ax.plot(bond_lengths, optimized_energies[0], ’o’, label=’UCCSD’, color=’red’)

ax.plot(bond_lengths, optimized_energies[1], ’x’, label=’UCCSD with Sampling Noise’, color=’blue’)

ax.plot(bond_lengths, exact_energies, ’-’, label=’Full-CI’, color=’black’)

ax.legend(frameon=False)

pyplot.show()
